# Altered Static and Dynamic Interhemispheric Resting-State Functional Connectivity in Patients With Thyroid-Associated Ophthalmopathy

**DOI:** 10.3389/fnins.2021.799916

**Published:** 2021-12-06

**Authors:** Wen Chen, Hao Hu, Qian Wu, Lu Chen, Jiang Zhou, Huan-Huan Chen, Xiao-Quan Xu, Fei-Yun Wu

**Affiliations:** ^1^Department of Radiology, The First Affiliated Hospital of Nanjing Medical University, Nanjing, China; ^2^Department of Endocrinology, The First Affiliated Hospital of Nanjing Medical University, Nanjing, China

**Keywords:** thyroid-associated ophthalmopathy, magnetic resonance imaging, resting-state, voxel-mirrored homotopic connectivity, dynamic analysis

## Abstract

**Purpose:** Thyroid-associated ophthalmopathy (TAO) is a debilitating and sight-threatening autoimmune disease that severely impairs patients’ quality of life. Besides the most common ophthalmic manifestations, the emotional and psychiatric disturbances are also usually observed in clinical settings. This study was to investigate the interhemispheric functional connectivity alterations in TAO patients using resting-state functional magnetic resonance imaging (rs-fMRI).

**Methods:** Twenty-eight TAO patients and 22 healthy controls (HCs) underwent rs-fMRI scans. Static and dynamic voxel-mirrored homotopic connectivity (VMHC) values were calculated and compared between the two groups. A linear support vector machine (SVM) classifier was used to examine the performance of static and dynamic VMHC differences in distinguishing TAOs from HCs.

**Results:** Compared with HCs, TAOs showed decreased static VMHC in lingual gyrus (LG)/calcarine (CAL), middle occipital gyrus, postcentral gyrus, superior parietal lobule, inferior parietal lobule, and precuneus. Meanwhile, TAOs demonstrated increased dynamic VMHC in orbitofrontal cortex (OFC). In TAOs, static VMHC in LG/CAL was positively correlated with visual acuity (*r* = 0.412, *P* = 0.036), whilst dynamic VMHC in OFC was positively correlated with Hamilton Anxiety Rating Scale (HARS) score (*r* = 0.397, *P* = 0.044) and Hamilton Depression Rating Scale (HDRS) score (*r* = 0.401, *P* = 0.042). The SVM model showed good performance in distinguishing TAOs from HCs (area under the curve, 0.971; average accuracy, 94%).

**Conclusion:** TAO patients had altered static and dynamic VMHC in the occipital, parietal, and orbitofrontal areas, which could serve as neuroimaging prediction markers of TAO.

## Introduction

Thyroid-associated ophthalmopathy (TAO), also known as Graves’ orbitopathy or endocrine exophthalmos, is a vision-disabling and disfiguring autoimmune disease, severely impacting the life quality, mental health, and socioeconomic status of the patients (Roos and Murthy, 2019; Taylor et al., 2020). Traditionally, the most common physical complaints about the disease are upper eyelid retraction, periorbital edema, exophthalmos, diplopia, and impaired visual function (Bahn, 2010). In recent years, however, additional emotional and psychiatric symptoms such as depression, emotional lability, memory deficits, and personality irregularities have attracted increasing attention of clinicians (Farid et al., 2005; Coulter et al., 2007; Silkiss and Wade, 2016). It has been reported that TAO not only restricted patients’ daily activities like reading, watching television, and enjoying free time, but also led to dysfunctions in social roles and impaired self-confidence associated with altered appearance (Zeng et al., 2019). It has also been observed that TAO patients had higher levels of anxiety and depression than people with other chronic diseases or facial disfigurements (Wickwar et al., 2015). A cohort study showed that TAO patients had a significantly higher risk of suicide (Ferlov-Schwensen et al., 2017). All these psychiatric signs suggested that TAO may be related to neuropsychic alterations rather than simple ophthalmic involvement (Bruscolini et al., 2018).

Previously, neuroimaging studies have revealed aberrant structural and functional brain alterations in TAO patients. Structural magnetization-prepared rapid gradient-echo (MPRAGE)-based research (Silkiss and Wade, 2016) found significant thinning of gray matter sheet in vision- and cognition-related brain areas. A combined voxel-based morphometry and diffusion tensor imaging study (Wu et al., 2020) demonstrated that TAO patients exhibited aberrant structural brain abnormalities corresponding to visual and cognitive deficits. A resting-state functional magnetic resonance imaging (rs-fMRI) study (Liu W. F. et al., 2019) showed decreased degree centrality in the cerebellum posterior lobe of TAO patients, which was correlated with anxiety and depression scores. Another rs-fMRI study (Tu et al., 2020) revealed that TAO patients with optic neuropathy had altered functional connectivity density (FCD) in the right orbital gyri of the frontal lobe and the left precuneus. However, these studies were still preliminary research using basic methodologies, which overlooked the interhemispheric functional integration and the temporal dynamic characteristic of brain activity over time. Further investigations with advanced technologies are needed to more fully elucidate the underlying neural mechanisms of TAO.

The human brain is a complex system involving distributed processing, and bilateral hemispheres are known to have robust homotopic connectivity (Zuo et al., 2010). Voxel-mirrored homotopic connectivity (VMHC) is a data-driven method of rs-fMRI, which computes the connectivity between each voxel in one hemisphere and its mirrored counterpart, quantifying an important feature of intrinsic brain functional architecture (Zuo et al., 2010). It measures integrity of information communication between hemispheres (Zuo et al., 2010). The method has been proven to be useful in detecting the interhemispheric functional connectivity alterations in various ophthalmic diseases, such as blindness, amblyopia, and glaucoma (Liang et al., 2017; Shao et al., 2018; Wang et al., 2018). However, whether the interactions between hemispheres are disturbed in TAO still remains unclear. Besides, mounting evidence has indicated that the resting-state functional connectivity is dynamic and varies during the whole scanning period (Preti et al., 2017). Thus, quantifying the temporal variability in functional connectivity metrics may help us comprehensively recognize the neural alterations and pathogenesis of diseases (Li et al., 2019). Given the previous clinical psychiatric findings and neuroimaging evidence, we hypothesized that TAO patients would have brain changes that could be detected by rs-fMRI with static and dynamic VMHC, especially in visual- and emotional-related areas.

Therefore, the purpose of this study was to verify our hypothesis by investigating the static and dynamic VMHC alterations in TAO patients. Moreover, as machine learning has received increasing attention and plays an important role in identifying potential neuroimaging biomarkers (Jia et al., 2020), we applied support vector machine (SVM), a supervised machine learning approach which allows individual-level classification (Orru et al., 2012), to test whether static and dynamic VMHC could be used to differentiate TAO patients from healthy controls (HCs).

## Materials and Methods

### Subjects

Twenty-eight consecutive TAO patients (15 females and 13 males, mean age 44.25 ± 12.71 years) were recruited from the department of endocrinology in our hospital. TAO duration was defined from onset of clinical manifestations such as upper eyelid retraction, lid lag, swelling, redness, and proptosis. Disease activity of TAO was assessed according to the modified 7-point Mourits’ clinical activity score (CAS) (Bartalena et al., 2021). Visual acuity measurement was also performed for each patient. Numerical values of the worse eyes for CAS and visual acuity were recorded. Concurrently, 22 age- and gender-matched HCs (12 females and 10 males, mean age 44.27 ± 12.75 years) were recruited in our study.

All subjects were in hematologically euthyroid state (TAO group: ≥3 months) when they participated in this study (Reference ranges: serum free triiodothyronine, 3.10–6.80 pmol/L; free thyronine, 12.00–22.00 pmol/L; thyroid-stimulating hormone, 0.270–4.200 mIU/L). The following exclusion criteria were applied to all subjects: (1) any evidence of other eye diseases (inflammation, orbital tumors, strabismus, amblyopia, cataracts, glaucoma, etc.); (2) history of eye surgery; (3) history of neurological or psychiatric illness (head injury, bipolar disorder, schizophrenia, etc.); (4) contraindications to MRI scan; and (5) alcohol or drug addiction. Comorbid anxiety and (or) depression symptoms were not considered as exclusion criteria if TAO was the primary clinical diagnosis. This study followed the tenets of the Declaration of Helsinki and was approved by the institutional ethical review board. Informed consents were obtained from all the subjects.

### Questionnaire Assessments

Life quality and neuropsychological assessments were conducted before MRI scan. The English version of Graves’ orbitopathy-specific quality of life (QoL) questionnaire was obtained from the EUGOGO website, and translated for TAO patient assessment (Lin et al., 2015). It contained two life quality subscales: visual functioning and appearance. Anxiety and depression symptoms were assessed in all subjects using the 14-item Hamilton Anxiety Rating Scale (HARS) and the 17-item Hamilton Depression Rating Scale (HDRS). Cognitive functions were assessed in all subjects using the Montreal Cognitive Assessment (MoCA).

### MRI Acquisition

All subjects were examined by using a 3.0-T MR imaging system (MAGNETOM Skyra; Siemens Healthcare, Erlangen, Germany) with a 20-channel head coil. Head motion and scanning noise were reduced by applying foam padding and earplugs to participants. The subjects were instructed to lie still in supine position with eyes closed, relaxing and staying awake. High-resolution sagittal structural T1-weighted images were acquired using MPRAGE sequence with the following parameters: repetition time (TR) = 1900 ms, echo time (TE) = 2.45 ms, flip angle = 9°, field of view (FOV) = 256 mm × 256 mm, matrix = 256 × 256, thickness = 1.0 mm, number of slices = 176, and voxel size = 1 mm × 1 mm × 1 mm. Functional images were collected axially by an echo planar imaging sequence with the following parameters: TR = 2000 ms, TE = 30 ms, flip angle = 90°, FOV = 240 mm × 240 mm, matrix = 64 × 64, thickness = 4.0 mm, number of slices = 35, and voxel size = 3.75 mm × 3.75 mm × 4 mm. The total scanning duration was 12 min and 26 s.

### Data Preprocessing

All the rs-fMRI data were preprocessed by using Data Processing Assistant for Resting-State fMRI advanced edition (DPARSFA) V4.4^1^ (Chao-Gan and Yu-Feng, 2010) based on SPM12^2^ (Ashburner, 2012). The preprocessing procedures were as follows: (1) converting Digital Imaging and Communications in Medicine (DICOM) files to Neuroimaging Informatics Technology Initiative (NIFTI) images; (2) removing the first 10 functional volumes to allow for equilibration of the magnetic field and for adaptation of the participants to the scanning environment; (3) slice timing correction for the remaining 230 fMRI images; (4) realignment for head motion correction; (5) reorientation of the structural and functional images; (6) segmentation of the structural images with the Diffeomorphic Anatomical Registration Through Exponentiated Lie Algebra (DARTEL) method (Ashburner, 2007) and generation of a group template; (7) spatial normalization to the Montreal Neurological Institute (MNI) template (resampling voxel size = 3 mm × 3 mm × 3 mm) using the segmented information from DARTEL; (8) spatial smoothing with a 6-mm full-width at half-maximum (FWHM) Gaussian kernel; (9) nuisance covariates regression [including the Friston 24-parameter model (Friston et al., 1996), signals of linear drift, white matter and cerebrospinal fluid]; and (10) temporal band-pass filtering (frequency range of 0.01–0.08 Hz). If the maximum value of the head translation (rotation) movement was over 2.0 mm (2.0°), the whole dataset of this participant would be discarded. In our study, all the subjects were preserved after head motion correction.

### Static Voxel-Mirrored Homotopic Connectivity Analysis

Static VMHC computations were also performed using DPARSFA V4.4. Firstly, a mean image was created by averaging the normalized T1-weighted images for all participants. Secondly, this image was averaged with its left–right mirrored version to generate a group-specific symmetrical template. The normalized T1 images were then registered to the symmetric template and applied to the non-linear transformation to the normalized functional images. Finally, for each participant, the homotopic connectivity was calculated as the Pearson’s correlation between the time series of each pair of mirrored interhemispheric voxels. Fisher *r*-to-*z* transformation was performed for the correlation coefficients to increase the normality of the distribution, and the VMHC *z*-maps were obtained (Zuo et al., 2010).

### Dynamic Voxel-Mirrored Homotopic Connectivity Analysis

Dynamic VMHC were computed with the Temporal Dynamic Analysis (TDA) toolkit based on DPABI V3.1^3^ (Yan et al., 2016, 2017). Sliding window-based analysis was applied to examine the whole-brain dynamic VMHC variability. In this study, a medium sliding window of 32 TR (64 s) and a shifting step size of 1 TR (2 s) were used, which could provide a good trade-off between the ability to resolve dynamics and the quality of connectivity estimation (Chen et al., 2020). For each subject, the remaining 230 time points after removing the first 10 time points was segmented into 199 windows. In each sliding window, VMHC was calculated by the same method used in the computation of static VMHC. The standard deviation of *z*-values at each voxel of all windows was calculated to depict the dynamic VMHC (Chen et al., 2020).

### Statistical Analyses

Demographic and clinical data were analyzed using the SPSS software (SPSS 22.0, Inc., Chicago, IL, United States). For continuous variables, two-sample *t*-tests (evaluating data with normal distribution) and Mann–Whitney *U* tests (evaluating data not normally distributed) were applied to compare the differences between TAOs and HCs. For categorical variables, Chi-square tests were used. The statistically significant threshold was set at *P* < 0.05 (Liang et al., 2017; Han et al., 2018).

For the static and dynamic VMHC values, statistical analyses were performed using SPM12. Two-sample *t*-test was performed to assess the group differences between TAOs and HCs, with age and gender controlled as confounding covariates. Statistical significance was based on a family-wise error (FWE) correction for multiple comparisons at the cluster level (*P*_FWE_ < 0.05) with a cluster-defining threshold of *P* < 0.001, in line with the current reporting guideline (Eklund et al., 2016). The surviving brain regions were mapped onto the cortical surfaces using the BrainNet Viewer software package^4^ (Xia et al., 2013).

The mean static or dynamic VMHC values in each significant cluster were extracted for each subject. After controlling the effect of age and gender, partial correlation analyses were performed to evaluate the relationships between static and dynamic VMHC values and clinical parameters in TAO group. Statistical significance was set at uncorrected *P* < 0.05, since the analyses were exploratory in nature.

### Support Vector Machine Analyses

To further evaluate whether the static and dynamic VMHC of significant clusters could be potential imaging biomarkers to identify TAOs from HCs, SVM analysis was performed using the LIBSVM software^5^ (Chang et al., 2000). Exploratory SVM analysis was conducted using a combination of these significant imaging features. For labeling of the subjects, TAO patients were labeled as 1, whilst HCs were labeled as −1. A linear kernel SVM was applied to perform the classifier training, in order to reduce the risk of overfitting and allow direct extraction of the feature weights (Pereira et al., 2009). The principle of SVM is to construct a separating hyperplane which maximizes the margin between the hyperplane and the support vectors (Pereira et al., 2009). The training model could be expressed as the following equation:


$$f\,\left(x\right)=w_{1}\,x_{1}+w_{2}\,x_{2}+\ldots +w_{\text{i}}\,x_{\text{i}}+b,$$

where *x*_i_ represents the *i*th feature vector, *w*_i_ represents the weight of the *i*th feature vector, *b* represents the bias, and *f*(*x*) represents the decision value. The predicted label would be 1 (TAO) if the decision value was positive, while −1 (HC) if the decision value was negative. Due to the limited number of samples, we employed a “leave-one-out” cross-validation (LOOCV) approach to evaluate the performance of the classifier (Pereira et al., 2009; Zhu et al., 2021). It was applied iteratively by leaving one subject out as the testing sample and using the remaining subjects as training samples. Receiver operating characteristic (ROC) curve analysis was used to examine the cross-validated performance of the SVM classification model. A non-parametric permutation test with 5000 permutations was applied to validate the significance of classification accuracy.

### Validation Analyses

To verify our finding of dynamic VMHC difference, we performed auxiliary analyses with different sliding-window lengths. In addition to 32 TR (64 s), another two window lengths [20 TR (40 s) and 50 TR (100 s)] were used to validate our results.

## Results

### Demographic and Clinical Characteristics

Table 1 presents the demographic and clinical characteristics of all participants. No significant difference was found in age (*P* = 0.907) or gender (*P* = 0.945) between the two groups. Compared with HCs, TAOs demonstrated significantly decreased visual acuity (*P* = 0.009). Moreover, TAOs had higher total scores of HARS (*P* < 0.001) and HDRS (*P* < 0.001), as well as lower total scores of MoCA (*P* < 0.001) than HCs.

**TABLE 1:** Demographic and clinical characteristics of TAO group and HCs.

Items	TAO group (*n* = 28)	HCs (*n* = 22)	*P*-value
Age (years)	44.25 ± 12.71	44.27 ± 12.75	0.907^a^
Gender (female/male)	15/13	12/10	0.945^b^
Disease duration (months)	18.39 ± 20.90	–	
CAS	2.54 ± 1.26	–	
Visual acuity	0.81 ± 0.25	0.98 ± 0.18	0.009^c^
QoL scores			
Visual functioning	61.95 ± 26.77	–	
Appearance	66.65 ± 19.70	–	
Total score of HARS	15.39 ± 8.51	2.68 ± 2.61	<0.001^a^
Total score of HDRS	15.25 ± 9.96	2.23 ± 1.93	<0.001^a^
Total score of MoCA	26.75 ± 2.66	29.09 ± 1.02	<0.001^a^

*Data were presented as mean ± standard deviation unless otherwise indicated.*

*CAS, clinical activity score; QoL, quality of life; HARS, Hamilton Anxiety Rating Scale; HDRS, Hamilton Depression Rating Scale; MoCA, Montreal Cognitive Assessment; TAO, thyroid-associated ophthalmopathy; HCs, healthy controls; n, number of subjects.*

*^a^P-value with Mann–Whitney test.*

*^b^P-value with Chi-square test.*

*^c^P-value with two-sample t-test.*

### Differences of Static and Dynamic Voxel-Mirrored Homotopic Connectivity

**FIGURE 1:**
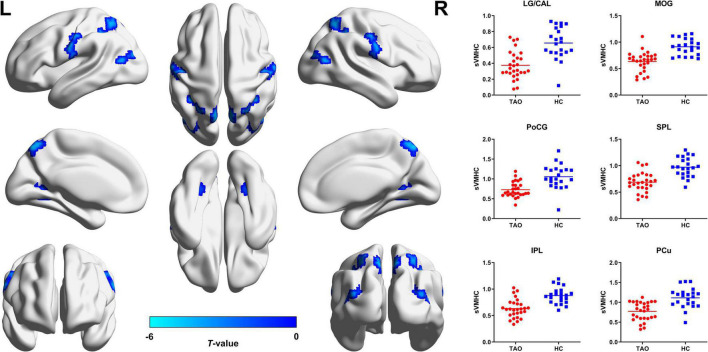
Brain regions with significant static VMHC difference between TAO group and HCs. Compared with HCs, TAO group showed significantly decreased VMHC values in LG/CAL, MOG, PoCG, SPL, IPL, and PCu (*P* < 0.05, cluster-level FWE corrected). The cold color denotes relatively lower values in TAO group, and the color bar indicates the *t*-value from two-sample *t*-test between TAO group and HCs. VMHC, voxel-mirrored homotopic connectivity; TAO, thyroid-associated ophthalmopathy; HCs, healthy controls; LG, lingual gyrus; CAL, calcarine; MOG, middle occipital gyrus; PoCG, postcentral gyrus; SPL, superior parietal lobule; IPL, inferior parietal lobule; PCu, precuneus; FWE, family-wise error; L, left; R, right; sVMHC, static VMHC.

For static VMHC, compared with HCs, TAO group showed significantly decreased VMHC values in lingual gyrus (LG)/calcarine (CAL), middle occipital gyrus (MOG), postcentral gyrus (PoCG), superior parietal lobule (SPL), inferior parietal lobule (IPL), and precuneus (PCu) (*P* < 0.05, cluster-level FWE corrected). Detailed information for brain regions with significant static VMHC difference between groups is shown in Table 2 and Figure 1.

**TABLE 2:** Brain areas with significantly different static and dynamic VMHC values between groups (*P* < 0.05, cluster-level FWE corrected).

Brain regions/conditions	BA	MNI coordinates			Cluster size (number of voxels)	*t*-Value
		X	Y	Z		
***Static VMHC***						
**TAO group < HCs**						
R/L LG/CAL	18/19	27/−27	−54	−3	120	−4.763
R/L MOG	19/37/39	36/−36	−78	18	52	−4.579
R/L PoCG	3/4/43	48/−48	−15	39	71	−4.574
R/L SPL	7	27/−27	−57	57	48	−5.621
R/L IPL	40	39/−39	−48	36	41	−4.897
R/L PCu	7	9/−9	−69	54	55	−4.777
***Dynamic VMHC***						
**TAO group > HCs**						
R/L OFC	11/47	30/−30	57	−6	20	5.551

*VMHC, voxel-mirrored homotopic connectivity; FWE, family-wise error; BA, Brodmann’s areas; MNI, Montreal Neurologic Institute; TAO, thyroid-associated ophthalmopathy; HCs, healthy controls; R, right; L, left; LG, lingual gyrus; CAL, calcarine; MOG, middle occipital gyrus; PoCG, postcentral gyrus; SPL, superior parietal lobule; IPL, inferior parietal lobule; PCu, precuneus; OFC, orbitofrontal cortex.*

**FIGURE 2:**
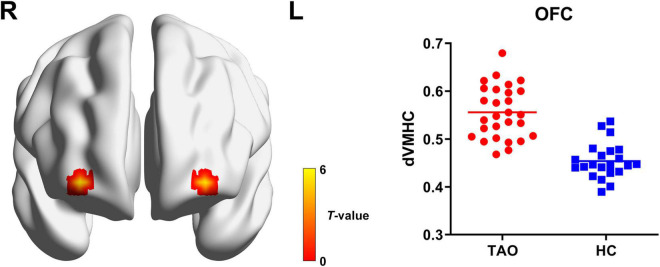
Brain regions with significant dynamic VMHC difference between TAO group and HCs. Compared with HCs, TAO group showed significantly increased dynamic VMHC variability in OFC (*P* < 0.05, cluster-level FWE corrected). The warm color denotes relatively higher values in TAO group, and the color bar indicates the *t*-value from two-sample *t*-test between TAO group and HCs. VMHC, voxel-mirrored homotopic connectivity; TAO, thyroid-associated ophthalmopathy; HCs, healthy controls; OFC, orbitofrontal cortex; FWE, family-wise error; R, right; L, left; dVMHC, dynamic VMHC.

For dynamic VMHC, TAO group demonstrated significantly increased dynamic VMHC variability in orbitofrontal cortex (OFC) than that of HCs (*P* < 0.05, cluster-level FWE corrected). Detailed information for brain regions with significant dynamic VMHC difference between groups is shown in Table 2 and Figure 2.

### Correlations With Clinical Measures

**FIGURE 3:**
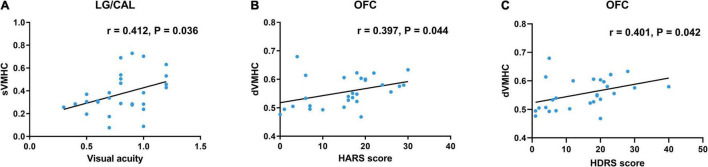
Scatter diagrams show the significant correlations with clinical and neuropsychological assessment in TAO patients. **(A)** Static VMHC in LG/CAL was positively correlated with visual acuity (*r* = 0.412, *P* = 0.036). **(B)** Dynamic VMHC in OFC was positively correlated with HARS score (*r* = 0.397, *P* = 0.044). **(C)** Dynamic VMHC in OFC was positively correlated with HDRS score (*r* = 0.401, *P* = 0.042). Age and gender were included as covariates. TAO, thyroid-associated ophthalmopathy; VMHC, voxel-mirrored homotopic connectivity; LG, lingual gyrus; CAL, calcarine; OFC, orbitofrontal cortex; HARS, Hamilton Anxiety Rating Scale; HDRS, Hamilton Depression Rating Scale; sVMHC, static VMHC; dVMHC, dynamic VMHC.

In TAO patients, static VMHC in LG/CAL was positively correlated with visual acuity (*r* = 0.412, *P* = 0.036) (Figure 3A). Moreover, dynamic VMHC in OFC of TAO patients was positively correlated with HARS score (*r* = 0.397, *P* = 0.044) (Figure 3B) and HDRS score (*r* = 0.401, *P* = 0.042) (Figure 3C). No significant correlation was found between static or dynamic VMHC and other clinical measures including disease duration, CAS, QoL scores, and MoCA score.

### Support Vector Machine Classification Results

**FIGURE 4:**
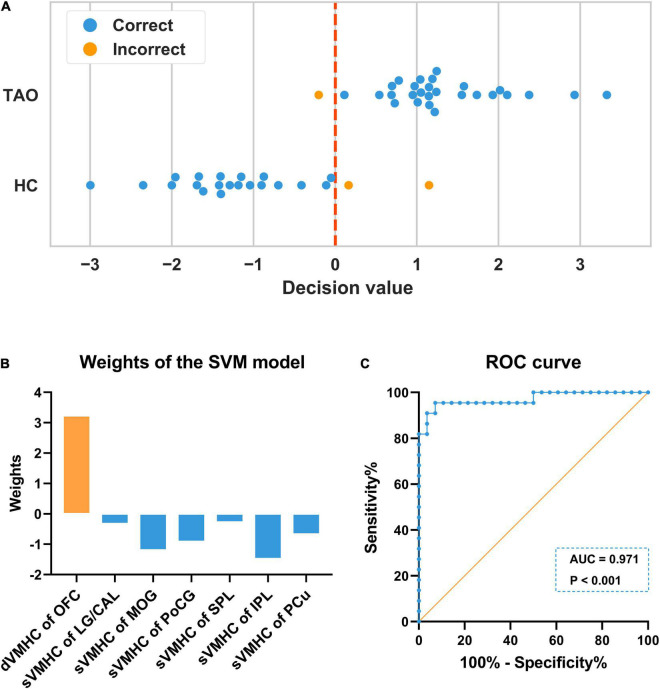
Illustration of the SVM classification results. **(A)** In each LOOCV, one subject’s data was left out as the testing sample, whose class label was then predicted with the training model based on the remaining subjects’ data. The predicted label would be 1 (TAO) if the decision value was positive, while –1 (HC) if the decision value was negative. The blue color denotes the sample was correctly classified, whilst the orange color denotes the sample was incorrectly classified. The final accuracy was 94% (47/50). **(B)** In the SVM model, dynamic VMHC of OFC exhibited higher weight than others in classifying the two groups. **(C)** The ROC curve showed that the model had good performance to distinguish TAOs from HCs, with an AUC of 0.971 (*P* < 0.001). SVM, support vector machine; LOOCV, “leave-one-out” cross-validation; TAO, thyroid-associated ophthalmopathy; HC, healthy control; VMHC, voxel-mirrored homotopic connectivity; dVMHC, dynamic VMHC; sVMHC, static VMHC; OFC, orbitofrontal cortex; LG, lingual gyrus; CAL, calcarine; MOG, middle occipital gyrus; PoCG, postcentral gyrus; SPL, superior parietal lobule; IPL, inferior parietal lobule; PCu, precuneus; ROC, receiver operating characteristic; AUC, area under the curve.

Our machine learning model showed good performance to distinguish TAOs from HCs, with an area under the curve (AUC) of 0.971 (*P* < 0.001). The average accuracy was 94% (*P* < 0.001, non-parametric permutation approach), whilst the sensitivity and specificity were 92.86% and 95.45%, respectively. In the SVM model, dynamic VMHC of OFC exhibited higher weight (3.201) than others (static VMHC of LG/CAL, −0.291; MOG, −1.165; PoCG, −0.882; SPL, −0.244; IPL, −1.449; and PCu, −0.640) in classifying the two groups. Detailed information of the SVM results is presented in Figure 4.

### Validation Results

The analyses using different sliding-window lengths supported our finding of dynamic VMHC difference. The significant cluster of increased dynamic VMHC variability in OFC also survived in the validation with sliding-window lengths of 20 and 50 TR (*P* < 0.05, cluster-level FWE corrected).

## Discussion

We applied a novel approach, by integrating machine learning with static and dynamic interhemispheric functional connectivity, to investigate the brain alterations of TAO. Our study had two main findings. First, TAO patients had significantly decreased static VMHC in occipital and parietal areas, as well as increased dynamic VMHC in OFC. Second, the machine learning model achieved great accuracy and efficacy in classifying TAOs and HCs. Moreover, the weight of dynamic VMHC in OFC exceeds those of static VMHCs, indicating the vital function of dynamic analysis. The present study provided robust evidence for interpreting the clinical-psychological symptoms, and expanded the understanding of brain functional changes in TAO.

The occipital cortex is well known to be responsible for visual processing, mainly associated with visual formation and visual perception activities (Yu et al., 2017). Specifically, CAL is the seat of primary visual cortex (V1) which receives direct visual inputs from the eyes *via* thalamic relays (Lu et al., 2019), LG is within the visual recognition network involved in encoding visual memories (Mao et al., 2020; Wang et al., 2020), and MOG is part of the dorsal visual areas which also plays a role in processing visual perception (Tu et al., 2013; Shao et al., 2019). Reduced VMHC in these regions have been reported in glaucoma (Wang et al., 2018), amblyopia (Liang et al., 2017), blindness (Shao et al., 2018), and globe injury (Ye et al., 2018), consistently interpreted as disturbed interhemispheric functional synchronization in visual cortices. Thus, our result of decreased static VMHC in LG/CAL and MOG also indicates disrupted interhemispheric functional coordination in visual cortical areas, and reflects impaired visual function in TAO patients. In previous structural imaging studies on TAO, significant thinning of gray matter sheet (Silkiss and Wade, 2016) and decreased fractional anisotropy (Wu et al., 2020) in the occipital cortex were also reported. Combined with the support of the positive correlation between the static VMHC in LG/CAL and visual acuity, we could further deduce that the visual cortex is impaired and the interhemispheric transmission of visual information may be reduced in TAO patients.

In addition to the expected alterations in the occipital cortex, decreased static interhemispheric functional connectivity was also found in the parietal areas in TAO cohort. The PoCG is the location of the primary somatosensory cortex (S1) and is responsible for sensory functions, including the encoding of touch and pain (Shao et al., 2018). Moreover, PoCG is thought to be part of the dorsal visual pathway and is involved in vision modulation (Longo et al., 2011; Chen et al., 2019). The SPL, located between the postcentral sulcus and occipital lobe, is also a part of visual pathway and participates in the visuo-motor coordination (Shao et al., 2018). Decreased functional connectivity between the left V1 and the bilateral SPL was previously found in patients with early blindness (Segal and Petrides, 2012). The IPL is located behind the lower part of the postcentral sulcus, and is the terminal of the dorsal stream (Huang et al., 2016). As a crucial component of the parieto-occipital pathway, IPL plays an important role in the encoding of spatial location and coordination of the visual-motor function, and is related to oculomotor, spatial attention and hand–eye coordination (Jiang et al., 2019). The PCu is the core of the default mode network (DMN), associated with various complex functions, such as recollection and memory, self-reflection, consciousness, and linking new information with experience (Cavanna and Trimble, 2006). In TAO, the structural-MPRAGE study by Silkiss and Wade (2016) showed significant thinning of gray matter sheet in PoCG, SPL, and PCu, which were highly consistent with the brain regions abnormalities found in this study. It is deemed that TAO patients not only undergo visual disturbances, but also may suffer from memory deficits, personality irregularities, and social isolations (Farid et al., 2005; Coulter et al., 2007; Jensen and Harder, 2011; Silkiss and Wade, 2016). Thus, integrating the neuroimaging findings with the cortical functions and the clinical psychiatric basis of the disease, we speculate that TAO might lead to the impairment of dorsal visual pathway and DMN, resulting in dysfunctions of oculomotor, visual attention, and cognition.

Another important finding of our study was the increased dynamic VMHC variability in OFC. The OFC plays a crucial role in emotional regulation and emotion-influenced decision-making (Zhang et al., 2020). Functional alterations within the OFC have been observed in various neuroimaging studies on anxiety- and depression-related disorders (Hahn et al., 2011; Li et al., 2019; Zhang et al., 2020). As we know, a proportion of TAO patients may show signs of anxiety and depression, and these symptoms can even be more prominent than in patients with other chronic diseases or facial disfigurements (Wickwar et al., 2015). Considering its significant correlations with HARS and HDRS scores, we believed that the aberrant dynamic VMHC variability in OFC may be related to the emotional disturbances in TAO. Moreover, the OFC is involved in the management of interpersonal relationships and social behavior (Blair, 2004), and this could be implicated in the social withdrawal and isolation in TAO patients (Jensen and Harder, 2011; Bruscolini et al., 2018). Furthermore, as a part of the prefrontal cortex, the OFC has fiber connections to all sensory areas and is involved in sensory information processing (Tu et al., 2020). Previous study by Bar et al. (2006) showed that the OFC was the origin of the top-down process in visual recognition. Therefore, we also hypothesize that the altered dynamic VMHC variability in OFC might influence the top-down transmission of visual information in TAO patients. The current finding of the OFC is in accord with the FCD-based research by Tu et al. (2020) which demonstrated abnormal FCD in the right OFC, suggesting that the OFC be a commonly involved area in TAO.

Interestingly, we observed that dynamic VMHC of OFC exhibited higher weight than the others in the SVM model, which hints the important role of dynamic analysis. To date, few studies have examined the neural alterations of diseases using both static and dynamic VMHC. The only available research that simultaneously utilized the two metrics was an investigation on subacute stroke (Chen et al., 2020). In their study, static VMHC identified more significant regions than dynamic VMHC, and regions showing significant differences in dynamic VMHC partially overlapped with those in static VMHC. They explained that static VMHC may be more sensitive in distinguishing brain areas demonstrating significant differences between patients and HCs, while dynamic VMHC may be more specific in distinguishing brain areas demonstrating significant intergroup differences. Our findings support their first viewpoint, as we also found that static VMHC could identify more significant regions. However, in our study, the region showing significant differences in dynamic VMHC (i.e., OFC) had no overlap with those in static VMHC. This finding is inconsistent with Chen et al. (2020), but in line with several studies using other static and dynamic metrics (Han et al., 2018; You et al., 2020). We hypothesize that this discrepancy might be associated with the distinct implications between static and dynamic analyses. Static metric is calculated based on full-length time series and mainly focuses on the metric itself (Liu H. et al., 2019), whereas dynamic metric captures the dynamics of brain activity patterns using sliding-windows, reflecting its temporal variability (Fu et al., 2018). Based on our results, we assume that TAO may only lead to excessive switching frequency of interhemispheric interaction in OFC, while no significant alteration of interhemispheric interaction in OFC could be induced. This assumption is supported by a study on schizophrenia (Damaraju et al., 2014), showing that pathological alterations could only affect some dynamic states. Of note, excessive variability, especially when it becomes chronic, is considered to have detrimental effects on cognitive functioning and emotional well-being (Christoff et al., 2016). In OFC, increased temporal variability has been observed in generalized anxiety disorder (Cui et al., 2020) and major depressive disorder with suicidal ideation (Li et al., 2019). Given the clinical psychiatric manifestations in TAO, it would be a reasonable finding that only dynamic VMHC was altered in OFC. Taken together, in our study, static VMHC identified disturbed interhemispheric interaction during the full-length time series in the occipital and parietal regions, while dynamic VMHC identified abnormally increased switching frequency of interhemispheric interaction in OFC. The difference may imply that neural alterations should not be delineated only based on single metric. Combining both static and dynamic metrics can provide more information, which enables us to better recognize the underlying neural patterns of diseases.

Currently, most neuroimaging studies conclude when they found differences with traditional inter-group statistical approaches. However, these findings could not be further applied at the individual level, which limited their translation into clinical application. The SVM is an analytical method which allows individual-level characterization, and therefore has high translational potential in a clinical setting (Orru et al., 2012). In this study, we tested the static and dynamic VMHC differences as features to discriminate TAOs from HCs through a linear SVM classifier, which achieved an AUC of 0.971 and an average accuracy of 94%. This finding suggested that static and dynamic VMHC differences could be potential neuroimaging markers for individualized differentiation between TAOs and HCs.

## Strengths and Limitations

Our study has strengths. The present work investigated the interhemispheric resting-state functional connectivity in TAO patients, by novelly combining static and dynamic VMHCs, integrated with a machine learning approach. To our knowledge, the present study is the first to explore the temporal dynamics of brain activities in TAO patients. Moreover, we firstly applied machine learning to neuroimaging investigations on TAO, and achieved an individual-level classification with high efficacy and accuracy. The findings expanded the current understanding of the neural alterations in TAO from the perspective of interhemispheric functional integration. More broadly, the results indicated the significance of combining static and dynamic neuroimaging metrics.

Several limitations should also be acknowledged. First, the sample size was limited due to the challenge of including euthyroid TAO patients as well as the strict criteria for controlling image quality and head motion. Further studies with larger sample sizes are warranted to verify the present results. Second, although significant static and dynamic VMHC differences were identified, the current findings were only derived from the single modality of rs-fMRI. Applying multiple neuroimaging modalities, such as white matter fiber-tracking, would be more meaningful to investigate the structural basis underlying the homotopic connectivity changes. Last, our cross-sectional design may limit the evaluations of brain alterations along with the development of the disease. Future prospective longitudinal research may be helpful to expand our understanding of this issue.

## Conclusion

Our study indicated that TAO patients had altered static and dynamic VMHC in the occipital, parietal and orbitofrontal areas, reflecting potential visual, emotional, and cognitive dysfunctions. These findings would enhance our current understanding of this disease, particularly in the neuropsychiatric aspect. The static and dynamic VMHC differences could serve as neuroimaging markers of TAO patients.

## Data Availability Statement

The raw data supporting the conclusions of this article will be made available by the authors, without undue reservation.

## Ethics Statement

The studies involving human participants were reviewed and approved by the Ethics Committee of the First Affiliated Hospital of Nanjing Medical University. The patients/participants provided their written informed consent to participate in this study.

## Author Contributions

HH, X-QX, and F-YW conceptualized and designed the study. WC, QW, LC, and JZ performed the MR scan. WC performed the MR data analyses and wrote the first draft. H-HC contributed to the diagnosis and clinical data collection. HH provided critical revisions of the draft. All authors approved the manuscript for submission.

## Conflict of Interest

The authors declare that the research was conducted in the absence of any commercial or financial relationships that could be construed as a potential conflict of interest.

## Publisher’s Note

All claims expressed in this article are solely those of the authors and do not necessarily represent those of their affiliated organizations, or those of the publisher, the editors and the reviewers. Any product that may be evaluated in this article, or claim that may be made by its manufacturer, is not guaranteed or endorsed by the publisher.

## Footnotes

1
http://rfmri.org/DPARSF

2
http://www.fil.ion.ucl.ac.uk/spm/software/spm12

3
http://rfmri.org/DPABI

4
http://www.nitrc.org/projects/bnv

5
http://www.csie.ntu.edu.tw/~cjlin/libsvm/
